# Morphological and physiological evidence of a synaptic connection between the lateral parabrachial nucleus and neurons in the A7 catecholamine cell group in rats

**DOI:** 10.1186/s12929-015-0179-2

**Published:** 2015-09-18

**Authors:** Chia-Yi Liu, Meng-Lam Lee, Chi-Sheng Yang, Chuan-Mu Chen, Ming-Yuan Min, Hsiu-Wen Yang

**Affiliations:** Department of Life Sciences, and Agricultural Biotechnology Center, National Chung Hsing University, Taichung, Taiwan; Department of Nursing, Jen-Teh Junior College of Medicine, Nursing and Management, Miaoli, Taiwan; Department of Biomedical Sciences, Chung Shan Medical University, 110, Chien-Kuo N. Rd, Sec. 1, Taichung, 402 Taiwan; Department of Medical Research, Chung Shan Medical University, Taichung, Taiwan; Department of Nursing, Hungkuang University, Taichung, Taiwan; Rong Hsing Research Center for Translational Medicine, and the iEGG Center, National Chung Hsing University, Taichung, Taiwan; Department of Life Sciences, College of Life Science, National Taiwan University, Taipei, Taiwan

**Keywords:** Lateral parabrachial nucleus, A7, Nociception, Catecholamine

## Abstract

**Background:**

The descending noradrenergic (NAergic) system is one of the important endogenous analgesia systems. It has been suggested that noxious stimuli could activate descending NAergic system; nevertheless, the underlying neuronal circuit remains unclear. As NAergic neurons in the A7 catecholamine cell group (A7) are a part of the descending NAergic system and the lateral parabrachial nucleus (LPB) is an important brainstem structure that relays ascending nociceptive signal, we aimed to test whether LPB neurons have direct synaptic contact with NAergic A7 neurons.

**Results:**

Stereotaxic injections of an anterograde tracer, biotinylated dextran-amine (BDA), were administered to LPB in rats. The BDA-labeled axonal terminals that have physical contacts with tyrosine hydroxylase-positive (presumed noadrenergic) neurons were identified in A7. Consistent with these morphological observations, the excitatory synaptic currents (EPSCs) were readily evoked in NAergic A7 neurons by extracellular stimulation of LPB. The EPSCs evoked by LPB stimulation were blocked by CNQX, a non-NMDA receptor blocker, and AP5, a selective NMDA receptor blocker, showing that LPB-A7 synaptic transmission is glutamatergic. Moreover, the amplitude of LPB-A7 EPSCs was significantly attenuated by DAMGO, a selective μ-opioid receptor agonist, which was associated with an increase in paired-pulse ratio.

**Conclusions:**

Taken together, the above results showed direct synaptic connections between LPB and A7 catecholamine cell group, the function of which is subject to presynaptic modulation by μ-opioid receptors.

## Background

Norepinephrine (NE) is an important neuronal modulator in the brain and plays significant roles in the regulation of many brain functions, including pain modulation [1]. Intrathecal injection of NE or α-2 receptor agonists results in dramatic analgesia in rats [2], the mechanisms underlying which have been shown to involve inhibitory effect of NE on nociceptive neurons located in the substantia gelatinosa area of the dorsal horn through activation of α2-adrenoceptors [1–3]. As there are no noradrenergic (NAergic) neurons in the dorsal horn of the spinal cord [4], it is generally believed that principal supply of NAergic innervation to the dorsal horn arises from the locus coeruleus (LC) (also referred to as the A6 catecholamine cell group) and the A7 catecholamine cell group (A7) located, respectively, in the dorsomedial and dorsolateral pons [5–8]. Direct stimulation of the A7 area results in an antinociceptive effect and the effect is blocked by intrathecal injection of α-2 receptor antagonists, showing that NAergic A7 neurons are indeed involved in intrinsic analgesia mediated by NE acting on α-2 receptors at the spinal cord level [9, 10].

The intrinsic NAergic pain regulatory system in the spinal cord has low tonic activity [11]. In support of this argument, recent studies of slice preparations have shown that NAergic A7 neurons spontaneously fire action potentials at a very low frequency (~0.5 Hz) when the fast synaptic transmissions are blocked [12, 13]. Furthermore, the spontaneous firing rate of NAergic A7 neurons is very sensitive to bath application of substance-P [12, 14] and GABA_B_ receptor antagonists [15], showing that synaptic drives are needed for operation of this system. In vivo studies have shown that peripheral noxious stimuli causes an early phase excitation of NAergic LC neurons, followed by a late phase of prolonged inhibition [16]; it also enhances NE release in the dorsal spinal cord [17, 18]. These observations suggest that ascending nociceptive signal might be a possible source of synaptic drive for activating NAergic neurons in the pons. Together with the fact that NE modulates nociceptive neurons in the spinal cord, it is likely that pontine NAergic neurons and dorsal horn neurons have reciprocal connections and that such reciprocal connections could function as a negative feedback system to inhibit the ascending nociceptive signal by causing NE release at the spinal level. However, the neuronal circuits that are responsible for the operation of this negative feedback control system remain unclear. For example, it is yet uncertain whether there are direct reciprocal projections between pontine NAergic and dorsal horn neurons, or relay by other nuclei in the brainstem is involved. A potential candidate that can relay the nociceptive signal to pontine NAergic neurons is the lateral parabrachial nucleus (LPB), which receives direct synaptic connections from dorsal horn neurons and transfers the nociceptive signal from peripheral to many pain-related areas in the brain [19–21]. Accordingly, the aim of this study is to test whether pontine NAergic neurons receive direct synaptic input from LPB.

## Methods

### Tracer injection

The use of animals in this study was in accordance with the rules for animal research of the Ethical Committee of Chung-Shan Medical University. Male Sprague-Dawley rats weighing 200–300 g were anaesthetized with 5 % isoflurane in pure oxygen. A small craniotomy was made, the dura was reflected, and a total of 0.3 μl of 10 % biotin dextran amine (BDA: MW 10,000; Invitrogen, Carlsbad, CA) in saline were injected via a 29-gauge stainless steel needle tilted 28 degrees to the vertical into the LPB of the left brainstem at the following coordinates: 5.30 mm posterior to the bregma, 1.9 mm lateral to the midline, 6.9 mm ventral to the cortical surface [22]. The stainless steel needle remained in place for 10 min after injection to minimize diffusion of the tracer along the needle tract. After completion of injection procedure, the needle was removed, the scalp was sutured, and the rats were replaced in their home cages.

### Immunohistochemical tissue processing

After a survival period of 3–4 days, the animals were deeply anesthetized with sodium pentobarbitone and perfused via the cardiac-vascular system with normal saline followed by fixative consisting of 4 % paraformaldehyde (Merck, Frankfurt, Germany) in 0.1 M phosphate buffer (PB), pH 7.4. The brains were then rapidly removed and placed in the same fixative at 4 °C for 3–4 h and stored overnight in cold (4 °C) 0.1 M PB. The brains were then transferred to 30 % sucrose in 0.01 M PB for cryoprotection. Serial saggital brainstem sections (50 μm thick) containing the A7 area and parabrachial nucleus were cut using a frozen sectioning technique. To visualize BDA, free-floating sections were incubated in ABC reagent (Vectastain ABC Peroxidase kit, Vector Labs, Burlingame, CA, USA) overnight at 4 °C. Following rinsing in 0.3 % Triton X-100 in phosphate buffered saline (TPBS) and in phosphate buffer (PB), BDA injection was visualized by the dark Nickel (Ni)- diaminobenzidine (DAB) reaction: 0.05 % DAB (DAB, Sigma, St. Louis, MO, USA) containing 0.01 % H_2_O_2_ and 0.04 % nickel ammonium sulphate in 0.1 M PB for 10 min. The reaction was terminated by extensive washes in PB. For visualization of noradrenergic neurons in the A7 area, sections were incubated in 2 % bovine serum albumin and 10 % normal goat serum for 1 h and subsequently in anti-rabbit tyrosine hydroxylase (TH; diluted 1:3000) overnight at 4 °C. The TH antibody was used here because we found that it stained dendritic structures better than that of the dopamine-β-hydorxylase antibody (DBH; Chemicon, Temecula, CA, USA) which was used for *post hoc* immunostaining of neurons after electrophysiology recording (see below). After rinsing with TPBS, the sections were incubated in biotinylated secondary antibody (diluted 1:200) at room temperature for 1 h. After several rinses in TPBS and in PB, immunoreactivity for TH was visualized by the red Nova Red reaction (Vector Labs) for 1–2 min at room temperature. For each brain, all sections were mounted on slides and alternate series were counterstained with cresyl violet to highlight cytoarchitonic divisions; the other sections were dehydrated in ethanol and coverslipped with DPX (Sigma).

### Electrophysiology

Sprague-Dawley rat pups of both sexes, aged 8–10 days, were used. They were anaesthetized with 5 % isoflurane in pure oxygen and decapitated. Their brains rapidly exposed and chilled with ice-cold artificial cerebrospinal fluid (ACSF) consisting of (in mM): 119 NaCl, 2.5 KCl, 1.3 MgSO_4_, 26.2 NaHCO_3_, 1 NaH_2_PO_4_, 2.5 CaCl_2_, and 11 glucose, oxygenated with 95 % O_2_ and 5 % CO_2_, pH 7.4. Sagittal brainstem slices (300 μm) containing the trigeminal motor nucleus (Mo5) and A7 area were cut using a vibroslicer (D.S.K. Super Microslicer Zero 1, Dosaka EM, Kyoto, Japan) and were kept in an interface-type chamber at room temperature (24–25 °C) for at least 90 min to allow recovery.

Slices were transferred to an immersion-type recording chamber mounted on an upright microscope (BX51WI, Olympus Optical Co., Ltd., Tokyo, Japan) and were continuously perfused with oxygenated ACSF at 2–3 ml/min. Neurons were viewed using Nomarski optics; those located about 200 μm rostral to the anterior border of Mo5 and having a large cell body (diameter about 20–25 μm) were considered to be NAergic A7 neurons [12–14] and were used for recordings (Fig. 3a). The patch pipettes, pulled from borosilicate glass tubing (1.5 mm outer diameter, 0.32 mm wall thickness; Warner Instruments Corp., Hamden, CT, USA), had a resistance of about 3–5 MΩ when filled with internal solution consisting of (in mM): 131 Cs-gluconate, 20 CsCl, 10 HEPES, 2 EGTA, 8 NaCl, 2 ATP, and 0.3 GTP; pH adjusted to 7.2 with CsOH. Recordings were made at room temperature (24–25 °C) with a patch amplifier (Multiclamp 700 A; Axon Instruments Inc.; Union City, CA, USA) in voltage-clamp mode. The membrane potential (Vm) was clamped at−70 mV and a voltage step of 5 mV was applied at 0.1 Hz throughout the recording to monitor serial resistances and the data were discarded if the values varied by more than 20 % of the original value, which was usually less than 20 MΩ. Signals were low-pass filtered at a corner frequency of 2 kHz and digitized at 10 kHz using a Micro 1401 interface running Signal software (Cambridge Electronic Design, Cambridge, UK) for episode-based capture recording. To elicit synaptic activity, a constant current pulse (25–500 μA; 100 μs) was delivered every 10 s through a bipolar stainless steel electrode (FHC, Bowdoinham, ME 04008 USA). To isolate excitatory postsynaptic currents (EPSCs) 1 μM strychnine plus 100 μM picrotoxin (Ptx) were added to ACSF. All data are presented as the mean ± standard error of the mean (SEM) and were compared using the paired *t* test. The criterion for significance was a *p* value < 0.05.

**Fig. 1:**
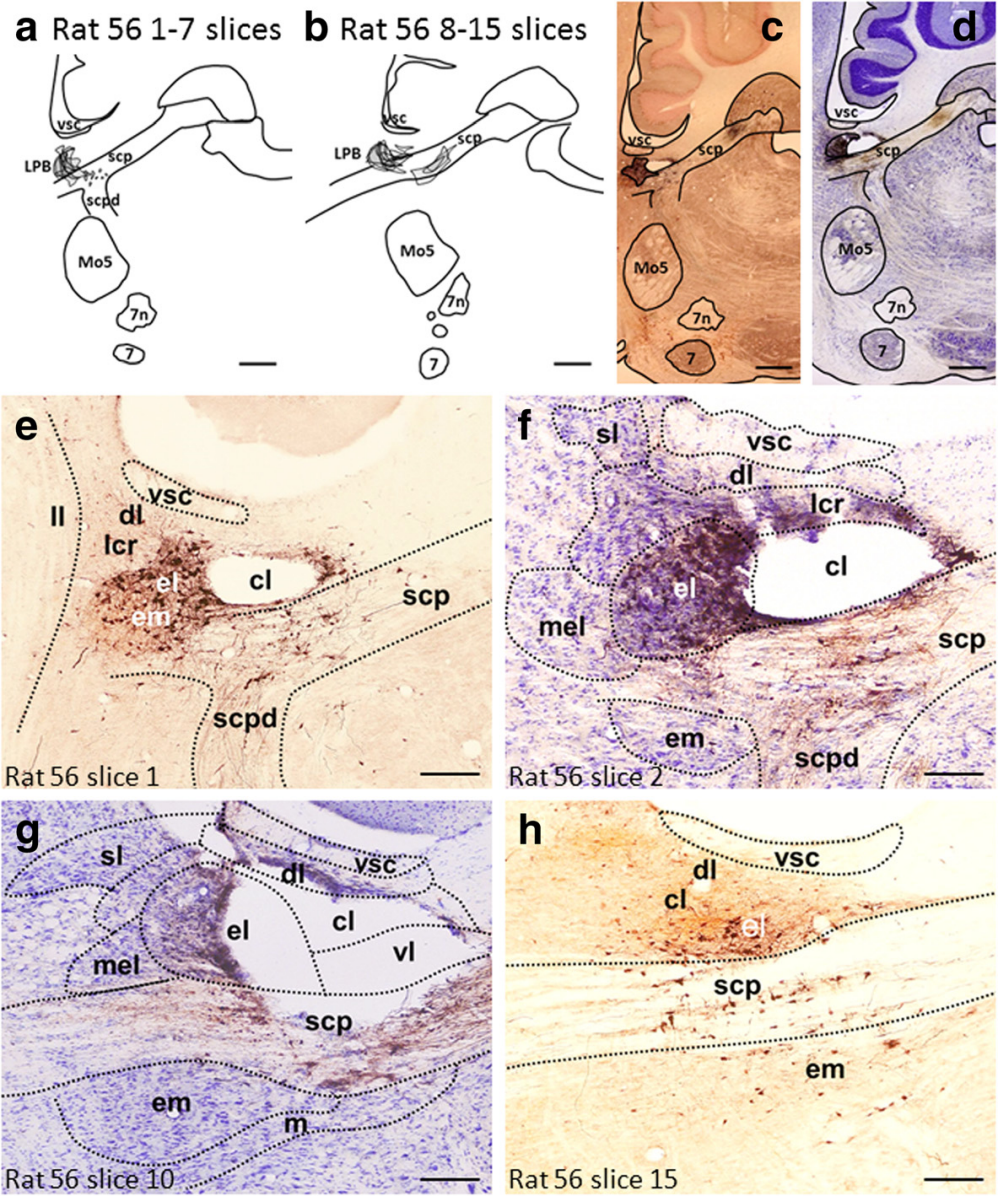
BDA injection sites. **a** & **b** Camera lucida drawings from saggital brainstem sections that contain a BDA deposit in the parabrachial nucleus (**a** & **b**). **c**-**g** Microphotographs of a BDA injection site are shown in low-power (**c**: Nova Red; **d**: Nissl-stained) and magnified views (**e** & **h**: Nova Red; **f** & **g**: Nissl-stained) The nomenclature for the divisions of subdivisions of the parabrachial nucleus was adapted from Sarhan et al. [21]. Note that the injection site is centered in the central lateral (cl) subnuclus of the parabrachial nucleus. cl, central lateral subnucleus; dl, dorsal lateral subnucleus; el, external lateral subnucleus; em, external medial subnucleus; lcr, lateral crescent subnucleus; ll, lateral lemniscus; LPB, lateral parabrachial nucleus; m, medial parabrachial nucleus; mel, external lateral subnucleus of the mesencephalic parabrachial nucleus; Mo5, trigeminal motor nucleus; scp, superior cerebellar peduncle; scpd, superior cerebellar peduncle, descending limb; sl, superior lateral subnucleus of the mesencephalic parabrachial nucleus; vl, ventral lateral subnucleus; vsc, ventral spinocerebellar tract; 7n, facial nerve; 7, facial nucleus. Scale bar = 500 μm (**a**-**d**), 150 μm (**e**-**h**)

In all experiments, 6.7 mM biocytin was routinely included in the internal solution to fill the recorded neurons (Fig. 3a). Neurons were filled by passive diffusion of biocytin from the patch pipette during the recording period, without application of current. After recording, the pipettes were withdrawn and the slices fixed overnight at 4 °C in 4 % paraformaldehyde (Merck) in 0.1 M phosphate buffer (PB, pH 7.4), then rinsed with PB several times, and subjected to immunohistochemistry (IHC) procedures without further sectioning. Briefly, the slices were rinsed in phosphate-buffered saline containing 0.03 % Triton X-100 (PBST), then incubated for 1 h at room temperature in PBST containing 2 % bovine serum albumin and 10 % normal goat serum. The slices were then incubated overnight at 4 °C in PBST containing a 1/1300 dilution of mouse antibody against rat DBH (Chemicon, Temecula, CA, USA) and a 1/200 dilution of avidin-AMCA (Vector Labs). After PBST rinses, the slices were incubated for 2 h with tetramethylrhodamine isothiocyanate (TRITC)- conjugated goat anti-mouse IgG antibodies (Jackson, Pennsylvania, USA) diluted 1/50 in PBST, then observed under a fluorescence microscope (Aioplan 2, Zeiss, Oberkochen, Germany) or a confocal microscope (Leica TCS SP5, Hamburger, Germany) for the identification of cell types (Fig. 1c-e).

All chemicals used to prepared ACSF and pipette solution preparation and Ba^2+^ were from Merck; Ptx, strychnine, and biocytin were from Sigma; and DL-2-amino-5-phosphonopentanoic acid (AP5), 6,7-dinitroquinoxaline- 2,3-dione (DNQX), DAMGO and naloxone were from Tocris-Cookson (Bristol, UK).

## Results

### BDA Injection sites

The localization of injection sites was examined and only experiments that had an injection site within the LPB area were accepted for further investigation. An example of an accepted injection site is shown in Fig. 1a, b. An overlay of camera lucida drawings of all sections with BDA deposits (Fig. 1a, b) shows that spreading of the injected BDA was restricted to the LPB area in this case. The injection sites (as indicated by the tissue damaged by the cannula tip) were centered on the central lateral (cl) LPB (Fig. 1c-h), and the injected BDA extended to the lateral crescent (lcr), external medial (em) and external lateral (el) subnuclei, as labeled neurons and neuritis were identified in these areas (Fig. 1e-h). Some fibers in the superior cerebellar peduncle (scp) were also labeled, but labeled neurons in the deep cerebellar nuclei were not observed. Similar injection site locations and trace spreading patterns were observed in the other animal. The injection sites of these two cases were compatible with previous studies [19, 21] and the observations described below were based on these two cases.

### Anterograde labeled axons in the A7 cell group

**Fig. 2:**
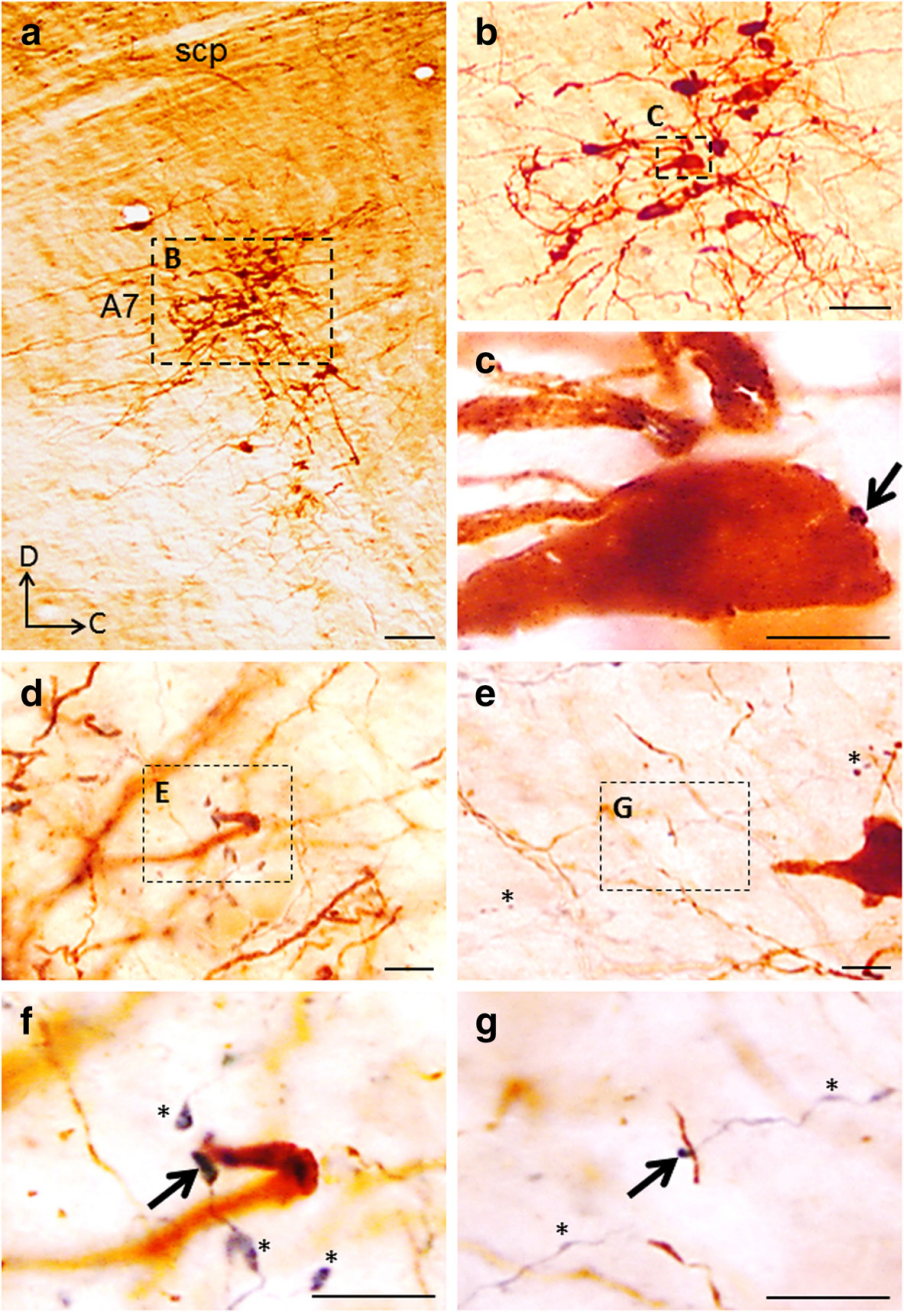
BAD-labeled axonal terminals in the A7 area. **a**. Light microscopic photograph of a rat brainstem section showing BDA deposit in the parabrachial nucleus and TH-ir neurons in the A7. Insert (**b**) shows a photograph of TH-ir neurons in the A7 with high power. Inserts (**c**), (**e**) and (**g**) show high power photographs of axonal terminals in the A7 area as indicated by dotted squares in the (**b**), (**d**) and (**f**), respectively. Note the terminals of BDA-labeled fibers with prominent en passant type varicosities (asterisks) and the contacts of terminals on large soma in (**c**), and dendrites in (**e**, **g**) as indicated by arrows. **d**, dorsal; scp, superior cerebellar peduncle. Scale bar = 100 μm (**a**), 50 μm (**b**), 10 μm (**c**-**g**)

In sections that comprise BDA injection sites and the trigeminal motor nucleus (Mo5), a cluster of TH-immunoreactive (TH-ir) neurons located rostral to the Mo5 could be labeled with an antibody to TH using Nova Red as the chromogen (Fig. 2a). This group of TH-ir neurons has soma of a multipolar shape (Fig. 2b) and is presumed to be NAergic A7 neurons. BDA deposits in the LPB produced antetrograde labeling of axons among cell bodies (Fig. 2a) and dendrites (Fig. 2d, f) of NAergic A7 neurons. These labeled axonal terminals with varicosity-like structures were stained dark blue by nickel intensified DAB histochemical procedures (see Fig. 2e, g). As the TH-ir elements were stained brown-red using Nova Red as the chromogen, they could be easily distinguished from the BDA-labeled axonal terminals. As can be seen (Fig. 2c, e, g), many swollen BDA-labeled varicosities and end-terminations could be clearly identified, some of these being in close proximity to soma (Fig. 2b, c), proximal and distal dendrites of NAergic A7 neurons (Fig. 2d-g).

### Characterization of LPB-A7 EPSCs

**Fig. 3:**
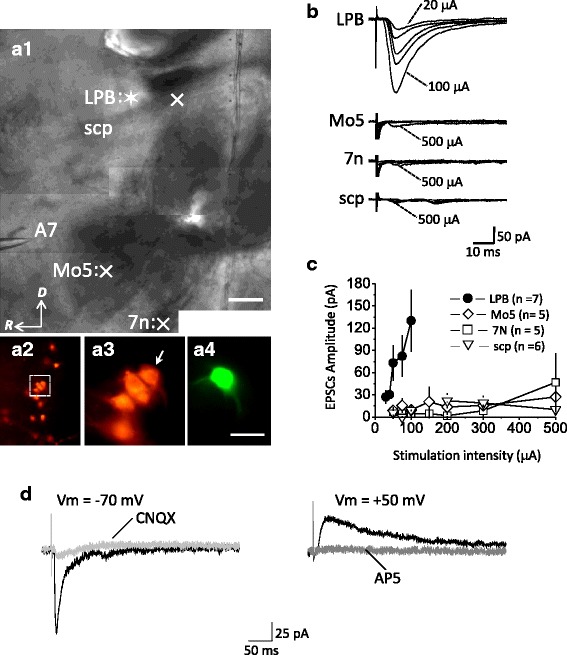
LPB-A7 EPSCs. **a**. Photographs of a Nomaskic image of a living saggital brainstem slice for electrophysiology recording (A1) and of a fluorescent image of *post hoc* immunostaining after recording (A2–A3). The asterisk marks the position of extracellular stimulation of the LPB and symbols × mark the positions of stimulation of the scp: superior cerebellar peduncle (*n* = 5), Mo5: trigeminal motor nucleus (Mo5) and 7n: the 7th (A1). A cluster of TH-ir neurons (A2, A3) was identified in A7: A7 catecholamine cell group (see glass pipette in A1), which was ~ 200 mm rostral to the anterior border of the Mo5. One of the TH-ir neurons (see arrow in A3) was recorded and filled with biocytin (A4). D: dorsal; *R*: rostral. **b**. Representative experiments show recording of EPSCs in NAergic A7 neurons responding to extracellular stimulation of the LPB (upper traces), Mo5 (middle upper traces), 7n (middle lower traces), and *scp* (bottom traces). **c**. Summarized results of the experiments shown in *B*. Note that, with a stimulating intensity < 100 μA, EPSCs were evoked only when the stimulating electrode was placed in the LPB. Parentheses indicate the number of experiments conducted for testing LPB (*n* = 7), Mo5 (*n* = 5), 7n (*n* = 50 and scp (*n* = 6) stimulation. **d**. Representative experiments show that LPB-A7 EPSCs were blocked by CNQX (Vm = -70 mV) and by AP5 (Vm = +50 mV). Scale bar = 250 μm (A1), 30 μm (A2–A4)

To further confirm that the above observed physical contacts between BDA-labeled varicosities and TH-ir soma and dendrites are functional synaptic contacts, we made whole cell recordings from NAergic A7 neurons in rats aged 8–10 days and examined whether extracellular stimulation to the LPB could evoke synaptic currents in NAergic A7 neurons. The electrophysiological criteria for recordings from NAergic A7 neurons have been described previously [12, 13]. Briefly, we made recordings in current-clamp mode and depolarizing/hyperpolarizing current pulses were injected to check the firing and membrane properties of the recorded neurons. Neurons that displayed neither a voltage sag nor rebound action potentials on injection of a hyperpolarizing current pulse, but displayed a voltage-dependent delay in initiation of the first AP on injection of depolarizing current pulses were used for subsequent voltage-clamp recordings (see [12, 13]). All of the recorded neurons showing above physiological criteria were further confirmed to be DBH-ir by *post hoc* IHC staining (Fig. 3a).

Under voltage-clamp recording with Vm clamped at−70 mV and blockade of GABAergic and glycinergic synaptic transmission by addition of 0.1 mM picrotoxin and 1 μM strychnine into the bath medium, extracellular stimulation of the trigeminal motor nucleus (Mo5), superior cerebellar peduncle (scp) and 7th nerve could not evoke detectable synaptic currents in NAergic A7 neurons until the stimulating intensity was increased up to 500 μA, when inward currents of small amplitude were elicited (Fig. 3b, c). In contrast, large and inward synaptic currents were evoked with a small stimulating intensity (threshold: 30 μA) in an intensity-dependent manner when the stimulating electrode was positioned in the LPB area (Fig. 3b, c). The synaptic currents evoked by LPB stimulation were blocked by bath application of 10 μM CNQX, a non-NMDA receptor blocker (Fig. 3d, left traces); clamping Vm at +50 mV in the subsequent recording revealed an outward current that was blocked by bath application of 50 μM AP5, a selective NMDA receptor blocker (Fig. 3d, right traces). These results show that the excitatory postsynaptic currents (EPSCs) evoked by LPB stimulation in NAergic A7 neurons (referred as LPB-A7 EPSCs) were glutamatergic, with glutamate acting at both non-NMDA and NMDA receptors. The latency, 10–90 % rise time, half width of LPB-A7 EPSCs was 3.3 ± 2.3 ms, 1.7 ± 0.2 ms and 8 ± 0.7 ms, respectively.

### Presynaptic modulation of μ-receptor on LPB-A7 EPSCs

**Fig. 4:**
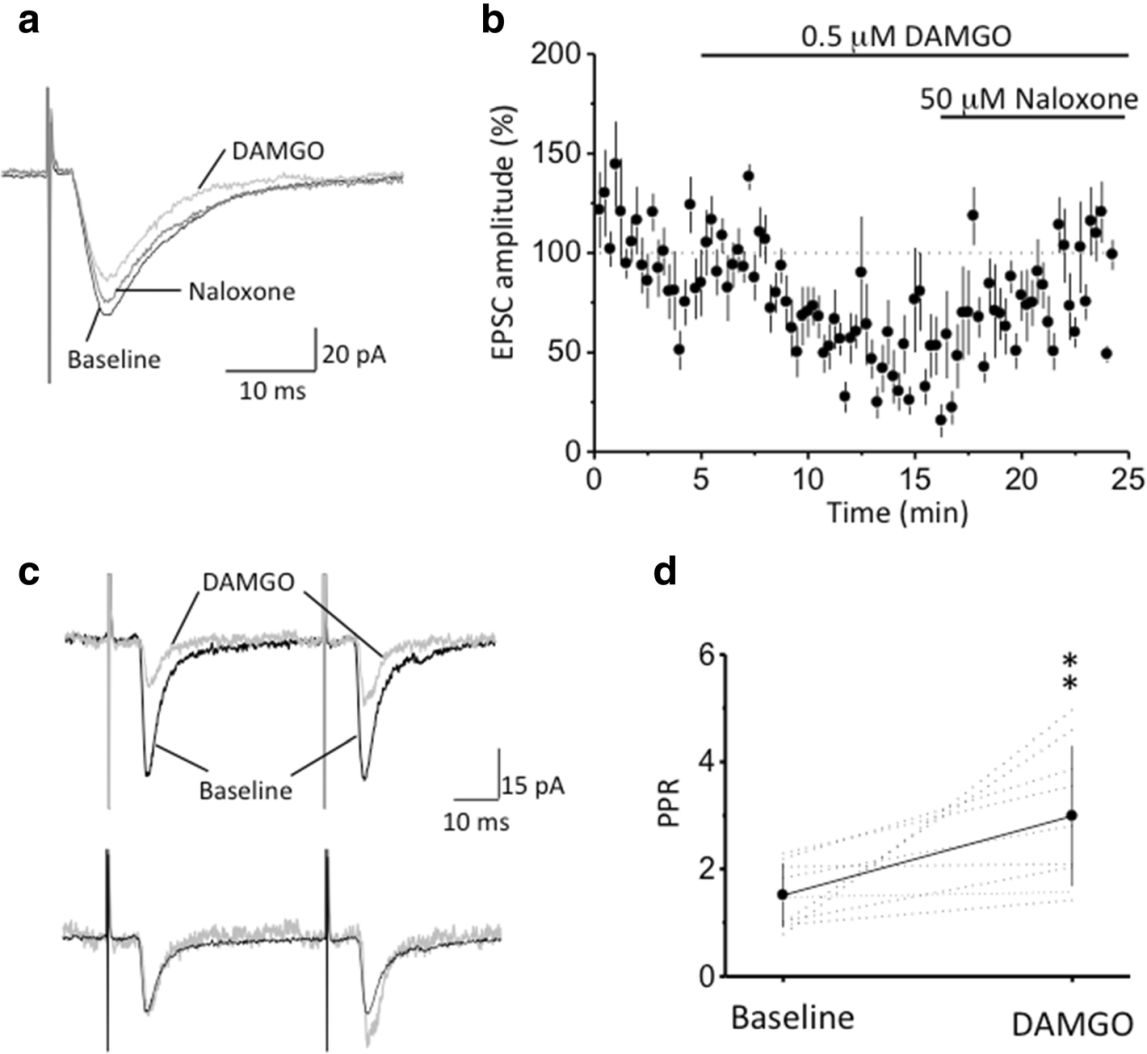
Presynaptic modulation of LPB-A7 EPSCs by μ−opioid receptors. **a**. Representative experiment showing that the amplitude of LPB-A7 EPSCs was attenuated by DAMGO, and the effect was reversed by subsequent application of naloxone. **b**. Summarized results of the experiments shown in **a**. The amplitude of the EPSCs was normalized to the averaged value of the baseline recording (see dotted line). **c**. Representative experiment showing the effect of DAMGO on the PPR of LPB-A7 EPSCs. The upper traces are overlays of raw data traces (black: before and gray: after DAMGO application); note that DAMGO attenuated EPSCs as shown above. The lower traces show normalization of the response evoked by the first pulse of the two traces shown above and reveal an increase in the PPR by DAMGO. **d**. Summarized results of the experiments shown in **c**. The dotted lines are the results of individual experiments and the symbol line and vertical line are the mean and standard error, respectively. Asterisks mark significance at *p* < 0.01

Given that LPB neurons have been shown to express large amounts of μ-opioid receptors at their axonal terminals to modulate the release of neurotransmitters [23, 24], activation of these presynaptic μ-opioid receptors at terminal from LPB are expected to have a presynaptic modulation on the evoked EPSCs if the activity was indeed resulted from LPB stimulation. We therefore examined the effect of μ-opioid receptor activation on the amplitude and paired-pulse ratio (PPR) of LPB-A7 EPSCs. Bath application of 0.5 μM DAMGO, a selective μ-opioid receptor agonist, significantly attenuated the amplitude of LPB-A7 EPSCs to 47.5 ± 12.6 % (*n* = 5 cells, *p* <0.01, paired-*t* test) of the baseline level (Fig. 4a, b). Subsequent application of 5 μM naloxone, a selective μ-receptor blocker, significantly reversed the effect of DAMGO; the EPSC amplitude was reversed to 86.7 ± 13.1 % of the baseline level (Fig. 4a, b), showing that effect of DAMGO application was due to activation of μ-receptors. In addition to reducing the EPSC amplitude, the effect of DAMGO was associated with an increase in PPR, an indicator of presynaptic effect [25]. The LPB-A7 EPSCs was evoked with a pair of pulses with 50 ms of inter-pulse interval and the PPR was measured as ratio of amplitude of EPSCs evoked by the second pulse to that by the first pulse. The PPR in control conditions was 1.51 ± 0.6 and was increased to 2.99 ± 1.32 (*n* = 9 cells, *p* <0.01, paired-*t* test) upon DAMGO application (Fig. 4c, d), indication a decrease in probability of glutamate release upon μ-receptor activation. These results show that activation of μ-receptors inhibits LPB-A7 synaptic transmission (Fig. 4a, b), which involved a presynaptic modulation (Fig. 4c, d). They further support the argument that the evoked EPSCs are specific to LPB stimulation.

## Discussion

The results of the studies described in this report provide morphological and electrophysiological evidence for the existence of a monosynaptic connection between neurons in the lateral parabrachial nucleus and noradrenergic neurons in the A7 catecholamine cell group. We have shown that LPB neurons project to the A7 and target on the soma, the proximal and distal dendrites of NAergic neurons. In addition, we have shown that excitatory synaptic responses evoked in the NAergic A7 neurons by extracellular stimulation of the LPB area were mediated by glutamate acting at both non-NMDA and NMDA receptors. The release of glutamate from the axonal terminal of the LPB neurons is modulated by μ-opioid receptors.

Our anatomical experiments of light microscopic observations show physical contacts of BDA-labeled varicosities on TH-ir elements, including soma and dendritic aborizations in the A7 and suggest the existence of direct synaptic connections between LPB neurons and NAergic A7 neurons. The reliability of such anatomical data depends critically on obtaining a precise BDA injection site in the LPB. Indeed, our conclusions were drawn from observations made from two animals in which the location of the tip of the injection cannula and deposits of the injected BDA were in the LPB area, though some contamination occurred in the adjacent white-matter structure, the superior cerebellar peduncle (scp; see Fig. 1). This observation of BDA-labeled fibers in the scp raises the possibility that some BDA-labeled buttons in A7 might come from cerebellar deep nuclei but not from the LPB. However, no previous study has ever demonstrated neuronal connection between the cerebellar deep nuclei and the A7 cell group and our electrophysiological results also disfavor this possibility (see below). In addition to those on TH-ir elements, there were also TH-ir varicosities (see asterisks in Fig. 2e)-g) making physical contacts with non-TH-ir postsynaptic elements. In addition to NAergic neurons, there are interneurons scattering over the A7 area and making synaptic contacts with NAergic neurons [12]. It has been suggested that some of these interneurons are GABAergic, and afferents inputs from the periaqueductal gray area (PGA) to the A7 area could indirectly regulate NAergic neurons through regulating of these GABAergic interneurons [26–30]. Accordingly, BDA-labeled varicosities without contacts with TH-ir postsynaptic elements may contact GABAergic interneurons in A7 and afferents from the LPB might also indirectly regulate NAergic A7 neurons through GABAergic interneurons.

Extracellular stimulation of the LPB area evoked EPSCs in NAergic A7 neurons with the intensity threshold being about 30 μA. In our data pool, EPSCs of large amplitude could be readily induced with a stimulating intensity lower than 100 μA; in contrast, within the same range of stimulating intensity (30–100 μA; see Fig. 3c), no detectable EPSCs could be induced when the stimulating electrode was positioned in the scp, Mo5 or 7th nerve. Since the distance of these structures to NAergic A7 neurons are similar to that of LPB to NAergic A7 neurons, especially scp is in vicinity of the LPB, these observations show that the spread of current delivered from the stimulating electrode with an intensity of < 100 μA was limited to the local area, so that LPB neurons were activated only when the stimulating electrode was positioned in the LPB but not in its vicinity. These observations also show no functional connection between cerebellar efferent and NAergic A7 neurons, therefore confirming our anatomical observations that DAB-labeled buttons found in A7 were indeed from the LPB but not scp. The inhibition of LPB-A7 EPSCs by DAMGO, a selective μ-opioid receptor, further supports the argument that EPSCs evoked by LPB stimulation were caused by glutamate release from terminals of LPB neurons, because LPB neurons have been shown to express μ-opioid receptors [23, 24]. Moreover, the effect of DAMGO on EPSC amplitude was associated with an increase in PPR, showing that μ-opioid receptors are located at the axonal terminal of LPB neurons and regulate glutamate release. The EPSCs evoked by local LPB stimulation were blocked by application of non-NMDA and NMDA receptor blockers; these results echo previous studies arguing that the majority of parabrachial neurons are glutamatergic [31, 32]. The features of short delay-latency, rapid and smooth rise phase, of PLB-A7 EPSCs suggest that PLB-A7 EPSCs are monosynaptic. In brain slice preparation at room temperature, the range of latency between electrical stimulation and onset of synaptic responses varies approximately from 2 to 4 ms among different synapses; for example, the latency of monosynaptic unitary EPSCs is described as 4 ms for mossy fibers on CA3 pyramidal neurons in hippocampus [33] and 3 ms for local inputs on stellate neurons in visual cortex [34]. Since our recording conditions are similar to these studies, namely using extracellular stimulation and making recording at room temperature, the latency of 3.3 ms for PLB-A7 EPSCs suggest that the transmission is monosynaptic.

## Conclusions

The above electrophysiological features together with anatomical data all support the argument that LPB input makes direct synaptic contacts with NAergic A7 neurons. Regarding to the physiological role, however, whether this connection is involved in regulation of nociception requires further evidences. This is because LPB receives not only ascending projections from nociceptive neurons in dorsal horn but also from the medial portion of the nucleus of the solitary tract, which conveys signals from many visceral receptors, such as baroreceptors and cardiopulmonary receptors, and from gustatory receptors [35–38]. Due to the limitation of experimental material (brain slice) used in this study, we were unable to specify whether the stimulated LPB neurons in brain slice or the BDA labeled LPB neurons in the tracing experiments were nociceptive. Nevertheless, as LPB is the major central target for the ascending nociceptive signal from the dorsal spinal cord [39–41] and NAergic A7 neurons forms a part of descending analgesia system by projecting their axonal terminals to the dorsal spinal cord, our results do suggest the possibility that noxious stimuli could excite NAergic A7 neurons, one of the component of descending NAergic system, through with the LPB-NAergic A7 connection; namely, LPB-NAergic A7 connection could function as a negative feedback control loop for pain regulation. Our results also show that LPB-NAergic synaptic transmission is regulated by presynaptic μ-opioid receptors. As neurons in the A7 catecholamine cell group (both NAergic and interneurons) receive enkephalin and other endogenous opioids projection from the rostroventromedial medulla and PGA [26, 27, 29], the efficiency and operation of LPB-NAergic A7 synapses, a possible feedback control loop for pain regulation, could be modulated by the other components of descending analgesic systems, such PAG and RVM.

## Abbreviations

cl: Central lateral subnucleus
dl: Dorsal lateral subnucleus
el: External lateral subnucleus
em: External medial subnucleus
lcr: Lateral crescent subnucleus
ll: Lateral lemniscus
LPB: Lateral parabrachial nucleus
m: Medial parabrachial nucleus
mel: External lateral subnucleus of the mesencephalic parabrachial nucleus
Mo5: Trigeminal motor nucleus
scp: Superior cerebellar peduncle
scpd: Superior cerebellar peduncle, descending limb
sl: Superior lateral subnucleus of the mesencephalic parabrachial nucleus
vl: Ventral lateral subnucleus
vsc: Ventral spinocerebellar tract
7n: Facial nerve
7: Facial nucleus
